# A New Subfamily of Glycoside Hydrolase Family 30 with Strict Xylobiohydrolase Function

**DOI:** 10.3389/fmolb.2021.714238

**Published:** 2021-09-07

**Authors:** Casey Crooks, Nathan J. Bechle, Franz J. St John

**Affiliations:** ^1^ Institute for Microbial and Biochemical Technology, Forest Products Laboratory, USDA Forest Service, Madison, WI, United States; ^2^ Engineering Mechanics and Remote Sensing Laboratory, Forest Products Laboratory, USDA Forest Service, Madison, WI, United States

**Keywords:** xylobiohydrolase, glycoside hydrolase, glycoside hydrolase family 30, AcXbh30A, *Acetivibrio clariflavus*, xylobiose, glucuronoxylan, *Hungateiclostridium clariflavum*

## Abstract

The *Acetivibrio* *clariflavus* (basonym: *Clostridium clariflavum*) glycoside hydrolase family 30 cellulosomal protein encoded by the Clocl_1795 gene was highly represented during growth on cellulosic substrates. In this report, the recombinantly expressed protein has been characterized and shown to be a non-reducing terminal (NRT)-specific xylobiohydrolase (*Ac*Xbh30A). Biochemical function, optimal biophysical parameters, and phylogeny were investigated. The findings indicate that *Ac*Xbh30A strictly cleaves xylobiose from the NRT up until an α-1,2-linked glucuronic acid (GA)-decorated xylose if the number of xyloses is even or otherwise a single xylose will remain resulting in a penultimate GA-substituted xylose. Unlike recently reported xylobiohydrolases, *Ac*Xbh30A has no other detectable hydrolysis products under our optimized reaction conditions. Sequence analysis indicates that *Ac*Xbh30A represents a new GH30 subfamily. This new xylobiohydrolase may be useful for commercial production of industrial quantities of xylobiose.

## Introduction

The most abundant hemicellulose, xylan, represents the second most plentiful terrestrial polysaccharide on earth (Saha, 2003). Given its abundance, it has received significant consideration by industry and academia for utilization through bioconversion to green chemicals and fuels (Naidu et al., 2018) as well as for contributions to human health and well-being (Ebringerova and Hromadkova, 1999; Vazquez et al., 2000; Aachary and Prapulla, 2011; Chen et al., 2012; EFSA Panel on Dietetic Products et al., 2018). There are numerous defined xylan subtypes distinguished by varying chain substitution characteristics of acetyl, α-l-arabinofuranosyl (Ara*f*), and α-1,2-linked 4-O-methyl-d-glucuronic acid (GA): substitution differences which are biomass source and plant tissue dependent (Biely et al., 2016; Busse-Wicher et al., 2016). Predominate xylan forms all have GA substitutions (Preston et al., 2003), and hydrolysis by endoxylanases results in neutral oligoxylosides and acidic aldouronates which contain at least a single GA substitution. Both neutral oligoxylosides and aldouronates have potential value directly as either a nutraceutical or a functional food ingredient, a pharmacologically active compound, or pharmacological precursors (Ebringerova and Hromadkova, 1999; Vazquez et al., 2000; Ebringerová et al., 2002; Ohbuchi et al., 2010; Penksza et al., 2018).

Generic endoxylanases cleave the β-1,4-xylan chain in accessible regions and are hindered by increased levels of xylan chain substitution. In contrast, several appendage-dependent endoxylanases have been described (St John et al., 2006a; Correia, Mazumder et al., 2011; Labourel, Crouch et al., 2016). These function to cleave the β-1,4-xylan chain at a defined site relative to a recognized substitution such as GA or Ara*f* and are relatively rare compared to enzymes with generic endoxylanase activity (St John et al., 2010; Aspeborg et al., 2012). Only glycoside hydrolase family 30 (GH30), subfamily 7 and 8 (GH30-7 and GH30-8) enzymes are biochemically shown to have GA-dependent function. The best characterized of these are the GH30-8 endoxylanases which cleave glucuronoxylans (GXns) to yield a limit product consisting of a population of reducing terminus penultimate GA-substituted aldouronates (Supplementary Figures S1A–C) (St John et al., 2006a; Vršanská et al., 2007; St John et al., 2011; Urbániková et al., 2011). These GH30-8 glucuronoxylanases establish a well-defined phylogenetic clade distributed primarily in saprophytic and plant pathogenic bacteria corresponding to Gram-positive and Gram-negative classes, respectively (St John et al., 2011). Other than a few characterized functional outliers (St John et al., 2014; St John et al., 2018), all studies of this GH30 subfamily confirm that it functions as strict GA-dependent glucuronoxylanases (Gallardo et al., 2010; Sainz-Polo et al., 2014; Maehara et al., 2018). The GH30-7 subfamily establishes a similarly well-defined phylogenetic clade but, in contrast, is represented almost entirely by fungi (St John et al., 2010). Given the subfamily designation, it is perplexing to find an enigmatic mixture of xylan hydrolyzing activities assigned to this GH30 subfamily. These activities include glucuronoxylanases with studies that indicate function nearly indistinguishable from bacterial GH30-8 glucuronoxylanases (Biely et al., 2014; Nakamichi et al., 2019b; Katsimpouras et al., 2019), reducing end xylosidase (REX) activity (Tenkanen et al., 2013; Nakamichi et al., 2019a), and non-reducing end xylobiohydrolase (XBH) activity (Šuchová et al., 2020b). This topic has recently been reviewed (Puchart et al., 2021). Many of these enzymes have significant secondary activities. In one recent case, a GH30-7 primary XBH was shown to have secondary endoxylanase activity (Šuchová et al., 2020b). Researchers are working to explain how so many representative functions are possible within a single subfamily grouping (Nakamichi et al., 2019a; Nakamichi et al., 2019b; Nakamichi et al., 2020a; Nakamichi et al., 2020b).

Recently, through proteomic analysis, two novel GH30 xylan active enzymes were identified as major constituents of the *Acetivibrio clariflavus* (basonym: *Clostridium clariflavum* and *Hungateiclostridium clariflavum* (Tindall, 2019)) cellulosome during growth on cellulosic substrates (Artzi et al., 2015). In this work, we characterize the biochemical function of the protein encoded by the Clocl_1795 gene of *A. clariflavus* and provide phylogenetic evidence that the encoded protein represents a new functional subfamily of GH30. This enzyme is shown to be a strict XBH, which, under optimal functional conditions, generates no other detectable hydrolysis products. Biophysical characterization of the catalytic domain of this novel enzyme, dubbed *Ac*Xbh30A (*Ac*Xbh30A-CD), shows a broad functional pH range, with increased stability and activity at higher pH values. In preparation of this manuscript, Šuchová et al. (2020a) published in consideration of the same enzyme. While there is some overlap in these works, our report offers substantial additional information regarding *Ac*Xbh30A. New information includes 1) detailed information regarding the purified protein, 2) optimum functional parameters and kinetic characterization of this purified enzyme under these optimum conditions, 3) a thorough treatment of phylogenetic relationships coupled to a broader analysis of subfamily function by screening three related enzymes, and 4) evaluation of the earlier reported endoxylanase activity (Šuchová et al., 2020a). *Ac*Xbh30A may be valuable in the specific production of X_2_ from lignocellulosic biomass and may work efficiently in this process when coupled to the selective processing of a hardwood GXn by a GH30-8 glucuronoxylanase, thereby specifically producing X_2_ and a two-component mixture of aldouronic acids which are readily separable from X_2_ (Supplementary Figures S1B–F).

## Materials and Methods

### Reagents

All reagents used for biochemical assays and enzyme studies were of the highest purity. Xylo- and cello-oligosaccharides and p-nitrophenol xylobioside (pNP-X_2_) were products of Megazyme (Bray, Ireland). Beechwood xylan (BeWX) and birchwood xylan (BiWX) were products of Sigma-Aldrich Co. (St. Louis, MO). The GH30-8 endoxylanase, *Ca*Xyn30B (Crooks et al., 2020), was used to prepare a beechwood xylan aldouronate ladder hydrolysate for XBH characterization (graphically depicted in Supplementary Figures 1A–C). Following limit hydrolysis, the reaction was precipitated (ppt) in 60% ethanol (EtOH) followed by centrifugation at 14k x g at room temperature for 20 min. The 60% EtOH supernatant was vacuum evaporated using a rotovap and recovered with a water rinse to a total recovered volume of 10 ml. Absolute EtOH was then added to 95% saturation and allowed to equilibrate for 20 min at room temperature. This was then centrifuged at 12.5k x g at room temperature for 20 min. The 60–95% EtOH cut pellet was washed (pellet was agitated) in 200 ml absolute EtOH, recovered by centrifugation as above, and dried at 70 C under house vacuum in a tared centrifugation bottle. The resulting pellet’s mass was determined and was readily soluble in water. The 60–95% EtOH precipitated fraction was redissolved to 20 mg/ml for use in biochemical reactions.

### Cloning, Expression, and Purification

An original expression construct of the Clocl_1795 gene (encoding UniProt accession No. G8LU16) of *A. clariflavus* was kindly provided by Professor Edward Bayer from the Weizmann Institute, Rehovot, Israel. This was subcloned to generate alternative protein expression products by PCR. These new DNA constructs were cloned into pBlueScript to verify the primary DNA sequence and then subcloned into pET28 between the NcoI and XhoI restriction enzyme sites. The DNA expression constructs of other enzymes studied in this work were prepared with the same approach but were obtained directly using Gibson Assembly (Gibson et al., 2009) of large synthetic DNA fragments (IDT, Coralville, IA) or subsequent subcloning manipulations of the assembled synthetic DNA (Supplementary Table S1).

*Ac*Xbh30A and the other proteins considered in this work were expressed using slightly modified auto-induction methods originally detailed by Studier (Studier, 2005). Selection was maintained using 50 μg/ml kanamycin, and the expression proceeded at 18 C for over 30 h. The cells were recovered by centrifugation (8k x g), and collected cell pellets were processed in preparation for immobilized metal affinity chromatography (IMAC) as previously detailed (St John et al., 2018) using sonication to lyse the cells. Following single-step elution IMAC, protein was desalted into 20 mM Tris base, pH 7.0 buffer using a Zeba desalting column (Fisher Scientific, Pittsburgh, PA) and analyzed by sodium dodecyl sulfate polyacrylamide gel electrophoresis (SDS-PAGE) (Laemmli, 1970) using a precast TGX 4–15% gradient gel (Bio-Rad, Hercules, CA). This manner of protein expression and purification was also used for the *Ac*Xbh30A protein homologs considered in this work. For *Ac*Xbh30A-CD, the step-elution IMAC product is referred to as the step elution product (SEP) throughout this work. For some studies, *Ac*Xbh30A was further purified using gradient IMAC, anion exchange, and gel filtration chromatography. This more thorough purification product is referred to as the gradient elution product (GEP). Briefly, IMAC was performed as above but for the use of a 0–500 mM imidazole elution buffer gradient over 20 column volumes. Following this, *Ac*Xbh30A-CD was exchanged into 25 mM Tris base of pH 8.0 containing 2 mM 2-mercaptoethanol and resolved using a Mono Q column (Cytiva, Marlborough, MA) with a 20-column-volume sodium chloride gradient. Peak fractions were combined and subsequently desalted into the gel filtration chromatography buffer composed of 20 mM HEPES, 250 mM sodium chloride, and 2 mM dithiothreitol of pH 8.0 for further purification using a Superdex column (Cytiva). Gradient separations were performed using a Bio-Rad BioLogic DuoFlow medium-pressure liquid chromatography system.

### Sequence Analysis and Phylogenetic Placement

BLASTp (Altschul et al., 1990) on the UniProt website^1^ (The UniProt Consortium, 2017) was used to collect amino acid sequences showing homology to *Ac*Xbh30A. Given the limited number of related sequences, NCBI BLASTp^2^ was further utilized to create a representative set of sequences (Johnson et al., 2008). Furthermore, the Carbohydrate-Active enZYmes (CAZy) database^3^ (Lombard et al., 2014) for glycoside hydrolase family 30 enzymes was utilized to collect CAZy-annotated enzymes for the GH30 subfamilies (St John et al., 2010). Preliminary amino acid sequence alignments were generated for consideration using the ClustalW (Larkin et al., 2007) alignment tool, and sequence dataset trimming was performed using MEGAX (Kumar et al., 2018). Trimmed sequence datasets were aligned using the MAFFT G-INS-1 progressive alignment strategy^4^ (Katoh and Toh, 2008), and phylogenetic trees were prepared in MEGAX using the maximal likelihood (ML) method with the LG substitution model (Le and Gascuel, 2008). Phylogenetic tree branch support values were obtained with 100 cycles of bootstrap analysis. Amino acid sequence identity levels of all-against-all of the collected sequence dataset were performed using the statistical sequence shuffling tool PRSS^5^ (Pearson, 2001).

### Functional Analysis of *Ac*Xbh30A

Thin layer chromatography (TLC) with the mobile phase chloroform: glacial acetic acid: water (6:7:1, v/v) was performed as previously described (St John et al., 2006b). TLC plate samples were spotted in 1 μl aliquots. A minimum of two ascensions were performed, and the plates were developed using N-(1-naphthyl)ethylenediamine dihydrochloride as previously described (Bounias, 1980). For initial TLC functional screening using the *Ac*Xbh30A-CD SEP or other protein homologs purified by IMAC, 10 μl reactions consisted of substrate polysaccharides at 10 mg/ml or oligosaccharides at 5 mM, 30 mM sodium acetate of pH 5.0, and 0.1 mg/ml bovine serum albumin (BSA). Later studies using the *Ac*Xbh30A-CD GEP preparation were performed as 200 μl reactions in a thermocycler to maintain reaction temperature. These reactions were at pH 4 sodium acetate, used the GEP at 2 μg/ml and 20 μg/ml, and were sampled at 15 and 300 min (5 h). From these reactions, a range of sample loads were used to adequately represent multicomponent mixtures.

HPLC studies were performed using an Agilent 1260 Infinity system with refractive index detection. Xylooligosaccharides (XOSs) were resolved on a Shodex SH1821 column (with a SUGAR SH-G guard column) at 60 C in the mobile phase 0.01 N H_2_SO_4_ at the flow rate of 0.8 ml/min. Initial HPLC reactions were 30 μl in volume, contained 30 mM sodium acetate of pH 5.0, 0.1 mg/ml BSA, and 5 mM X_4_, and were initiated following equilibration to 40 C by addition of enzyme to 5 μg/ml and allowed to react for 20 min before heat inactivation at 95 C for 5 min. Stopped reactions were centrifuged for 5 min, and the supernatant was transferred for analysis by HPLC. Later studies used only 0.02 mg/ml BSA, and a four-component HPLC-compatible buffer (FAMM buffer) was developed to study optimum reaction pH over what was determined to be a broad functional range. FAMM buffer consisted of 30 mM each of sodium formate, sodium acetate, MES (2-(N-morpholino)ethanesulfonic acid), and MOPS (3-(N-morpholino)propanesulfonic acid) which should effectively buffer from pH 2.5 to 8.0. Single-component buffers including glycine, acetate, and Tris base used at 30 mM were also tested to validate results obtained using the FAMM buffer. All buffer compositions were pH adjusted with either 6N HCl or 1N NaOH. Heat inactivation of *Ac*Xbh30A-CD under more acidic conditions failed to fully inactivate *Ac*Xbh30A-CD, and a fix was found to be pH neutralization prior to heat inactivation. Specific activity analysis of oligoxylosides was performed as above, but using pH 4.0 sodium acetate, XOS at 10 mM, at a reaction temperature of 65 C for 10 min. Equilibrated reaction mixtures were initiated by addition of *Ac*Xbh30A-CD GEP to a final reaction concentration of 500 ng/ml. HPLC samples were analyzed a minimum of three times to obtain average values, and assays were performed a minimum of two times.

The substrate pNP-X_2_ was used for kinetic evaluation of *Ac*Xbh30A-CD GEP. This was performed in the same conditions described for the specific activity determination with XOS with the GEP preparation of *Ac*Xbh30A-CD except for an increase in volume to 100 μl. The reaction was stopped and developed by addition of 300 μl of 200 mM sodium carbonate. Samples were read at 405 nm, baseline corrected by subtraction of a no enzyme control, and adjusted for the development dilution of pNP. This was then converted to concentration values using the pNP mM extinction value of 18.4 (mM^−1^ cm^−1^). Kinetic analysis was performed using a range of pNP-X_2_ concentrations and the resulting kinetic constants determined from three replicate studies. A least-squares fit of the non-linear Michaelis–Menten enzyme kinetics model in GraphPad Prism 8 (GraphPad Software, San Diego, CA) was used to obtain kinetic values of the three curves as a group. The error is reported as the standard error of parameters, and the standard error of fit was 2.532 U/mg. The primary hydrolysis product X_2_ was considered for its role in inhibition of *Ac*Xbh30A. Duplicate substrate response curves were generated for no added X_2_, 0.5 mM X_2_, and 1 mM X_2_, and only a single curve was generated for 2 mM X_2_ and 4 mM X_2_. A robust fit of the non-linear competitive enzyme inhibition model in GraphPad Prism 8 was used to obtain the inhibition constant (*K*_*i*_).

## Results and Discussion

### *Ac*Xbh30A DNA Cloning, Protein Expression, and Initial Purification

The *Ac*Xbh30A enzyme was first identified following its proteomic detection as a major protein component of the *A. clariflavus* cellulosome during cultivation on cellulosic substrates (Artzi et al., 2015). Activity screening as part of that research determined *Ac*Xbh30A to have an unknown xylanase activity. The primary amino acid sequence for *Ac*Xbh30A consists of an N-terminal secretion leader sequence, a GH30 catalytic center, and a C-terminal type I dockerin domain. The original expression plasmid (Artzi et al., 2015) encoded the full *Ac*Xbh30A protein with an N-terminal His-tag in place of the predicted secretion signal sequence. The product of this original *Ac*Xbh30A expression plasmid was expected to yield full-length *Ac*Xbh30A with an N-terminal His-tag. However, upon IMAC purification and SDS-PAGE analysis, the preparation yielded two bands (Supplementary Figure S2). The minor band ran to the size expected for the full *Ac*Xbh30A protein, while the major band ran slightly smaller. Suspecting this to have resulted from proteolytic processing, we generated two constructs of *Ac*Xbh30A by cloning with a His-tag appended to the C-terminus of a full-length construct and also to a catalytic domain (CD) only construct. It was later found, following the publication by Šuchová et al. (2020a), that the pair of bands we observed by SDS-PAGE of the original expression construct is similar to that seen on the SDS-PAGE provided by NYZTech^6^ from where that group obtained *Ac*Xbh30A for study.

While this work primarily focuses on the characterization of *Ac*Xbh30A-CD, both *Ac*Xbh30A and *Ac*Xbh30A-CD constructs expressed to high levels. Initial IMAC purification of both forms resulted in a single prominent band of the expected molecular weight by SDS-PAGE and was judged to be greater than 95% pure (Supplementary Figure S2). Analysis of these enzyme forms by modified SDS-PAGE sample preparation showed that the *Ac*Xbh30A (dockerin-containing form) expression product is a disulfide-mediated dimeric product. Since this dimerization is not observed with *Ac*Xbh30A-CD and almost completely present with *Ac*Xbh30A which contains the C-terminal dockerin domain, we conclude the disulfide bond formation must involve Cys516 located in the dockerin domain. The artificial nature of high-level protein overexpression precludes prediction of any biological role for this disulfide bond occurrence. Cys516 is not conserved among other bacterial GH30 XBHs brought forth by these studies (Figure 5).

### Functional Characterization and Optimization of *Ac*Xbh30A Activity

**FIGURE 1:**
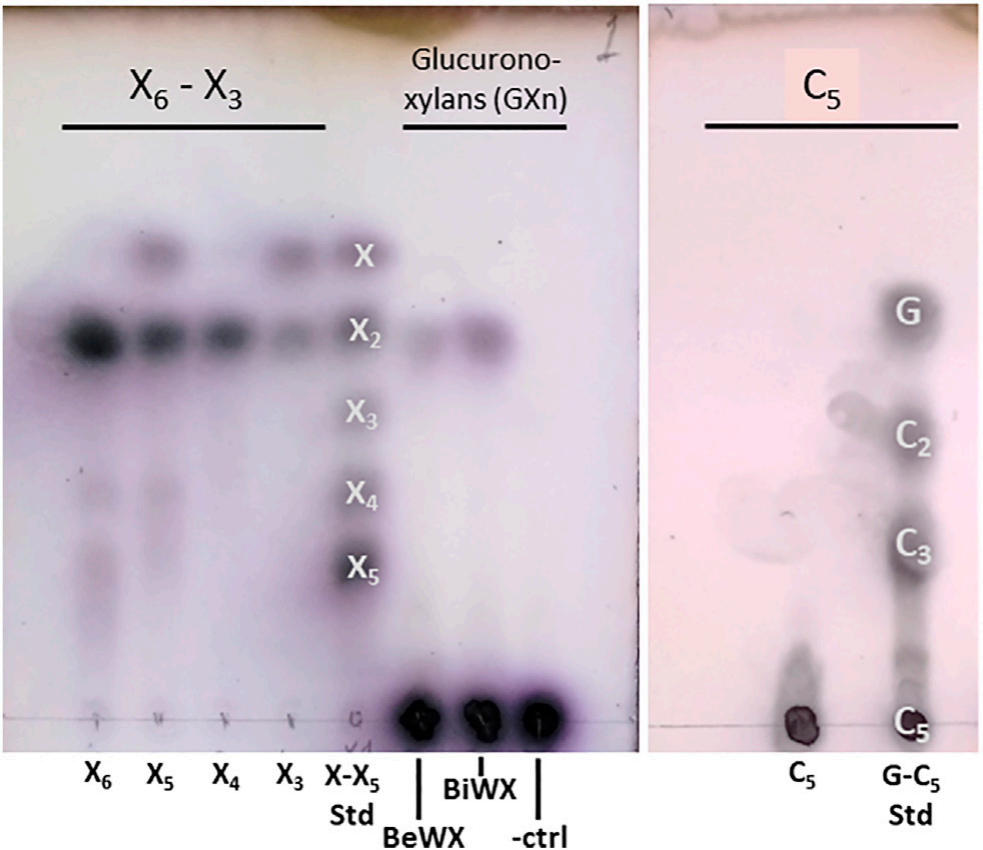
TLC showing *Ac*Xbh30A-CD hydrolysis of XOS, two common GX_n_ types, and cellopentaose. The results indicate that only a small amount of X_2_ is liberated from polymeric GX_n_, while hydrolysis of even-numbered XOSs yielded only X_2_ and hydrolysis of odd-numbered XOSs yielded X_2_ and xylose. Importantly, using the same TLC method, no analogous activity could be detected for the *Ac*Xbh30A-CD hydrolysis of cellopentaose (C_5_), indicating that the C6 position is not accommodated in the -1 and/or -2 subsites that establish the X_2_ binding pocket. Samples and standards for the xylan-based assay include xylose (X) through xylopentaose (X_5_) with beechwood glucuronoxylan (BeWX) and birchwood glucuronoxylan (BiWX) reactions as well as a BeWX no enzyme control (-ctrl). For C_5_ hydrolysis, analysis included glucose (G), cellobiose (C_2_), cellotriose (C_3_) and cellopentaose (C_5_) as standards.

Initial biochemical studies were performed using the *Ac*Xbh30A SEP preparation. Numerous biomass-derived polysaccharides were tested for TLC observable activity (data not shown). For both enzyme constructs, only xylobiose (X_2_) was observed as a product from the hydrolysis of GXn. Hydrolysis of even-numbered XOSs yielded only X_2_, and hydrolysis of odd-numbered XOSs yielded X_2_ and xylose (Figure 1). Hydrolysis of cellopentaose did not release a smaller hydrolysis product, indicating that the C6 (CH_2_OH) of the glucose is not accommodated in the substrate binding pocket.

Following function determination, XBH activity was studied by following hydrolysis of X_4_ to X_2_ by HPLC. Initial reaction optimization showed that *Ac*Xbh30A-CD had a greatly reduced activity when not supplemented by BSA (at 0.1 mg/ml), while the dockerin-containing *Ac*Xbh30A enzyme form did not appear to benefit from BSA inclusion. For the *Ac*Xbh30A-CD SEP, MgCl_2_ at 0.1 mM did not benefit activity and CaCl_2_ at this concentration resulted in a 9% reduction in activity. Given the BSA activity dependence of the CD form, a BSA titration study was performed. Addition of BSA from 0.005 to 0.2 mg/ml resulted in increasing activity to form a horizontal asymptote. From this observation, BSA was included in all future reactions at a level of 0.02 mg/ml.

**FIGURE 2:**
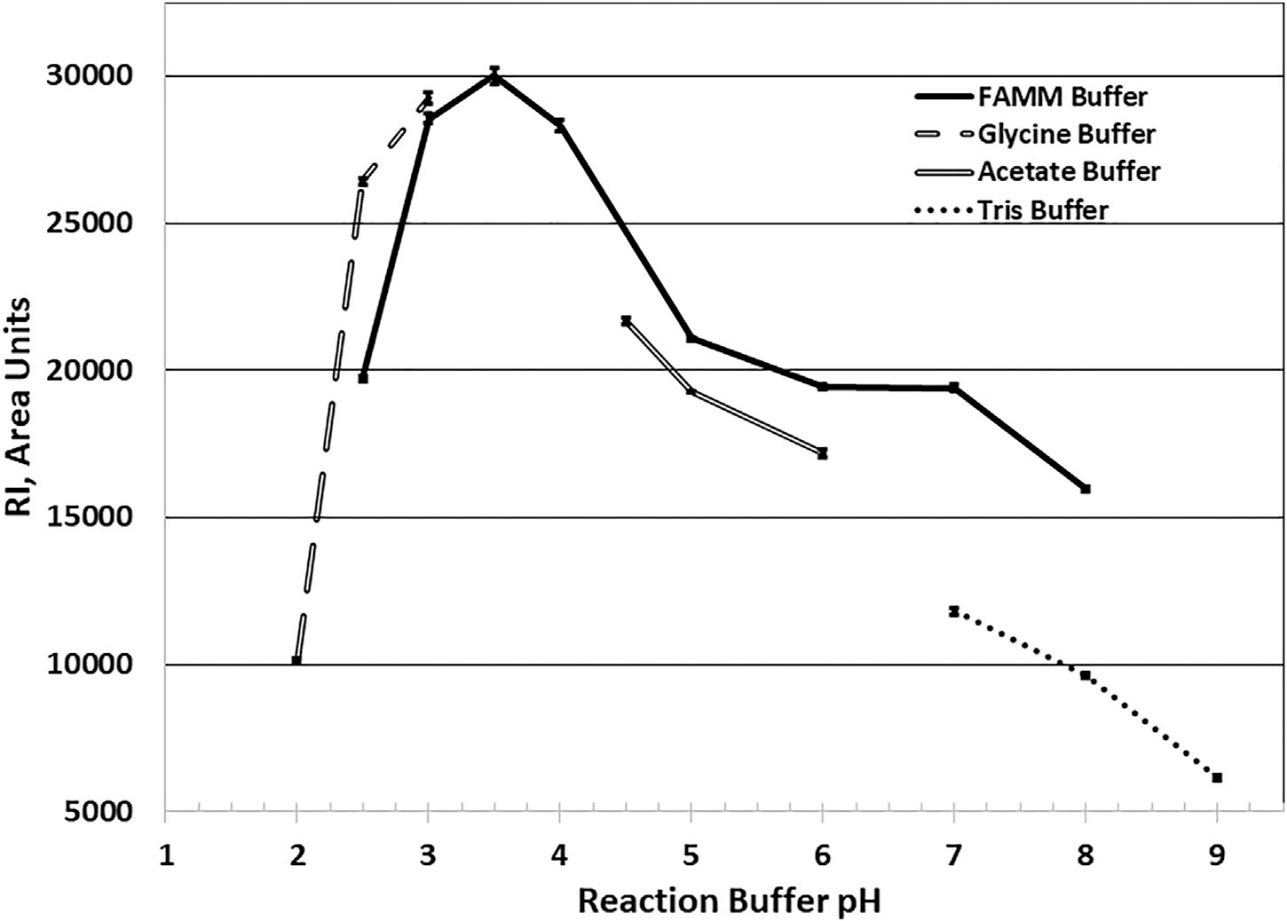
*Ac*Xbh30A-CD SEP X_4_ hydrolysis reaction optimum pH determination. All buffers were used at 30 mM final concentration for each buffer component, and the reaction was maintained at 40 C for 20 min with BSA included at 0.02 mg/ml. The findings show that, at the selected reaction temperature of 40 C, the optimum reaction pH using the FAMM buffer system is approximately 3.5. By extrapolation of the curve, the optimal pH is mirrored using the single-component buffers glycine and acetate. The use of Tris buffer to verify pH activity dependence at the higher pH conditions yielded significantly lower activity.

Optimum reaction pH studies indicated that *Ac*Xbh30A-CD was functional over a broad range. To accommodate this, a four-component buffer system (FAMM buffer) was developed to be compatible with the HPLC system conditions. In these HPLC studies, through serial sample injections, it was observed that, at pH 2.5 and 3.0 reaction conditions, *Ac*Xbh30A-CD was partly resistant to inactivation at the subsequent 95 C heat treatment. Although several approaches were successfully shown to inactivate *Ac*Xbh30A-CD while under these reaction conditions, simple pH quenching with a 2x dilution of 150 mM Tris base of pH 8.0 effectively allowed for complete *Ac*Xbh30A-CD inactivation at 95 C. Using the FAMM buffers in 40 C reactions, the *Ac*Xbh30A-CD SEP observed optimum activity at pH 3.5, maintained about 66% of its optimum activity at pH 2.5 and pH 6, and retained greater than 50% of its optimum activity at pH 8 (Figure 2). Other single-component buffers closely mirrored the FAMM pH curve, except for Tris buffer which yielded significantly lower activity at the higher pH conditions.

**FIGURE 3:**
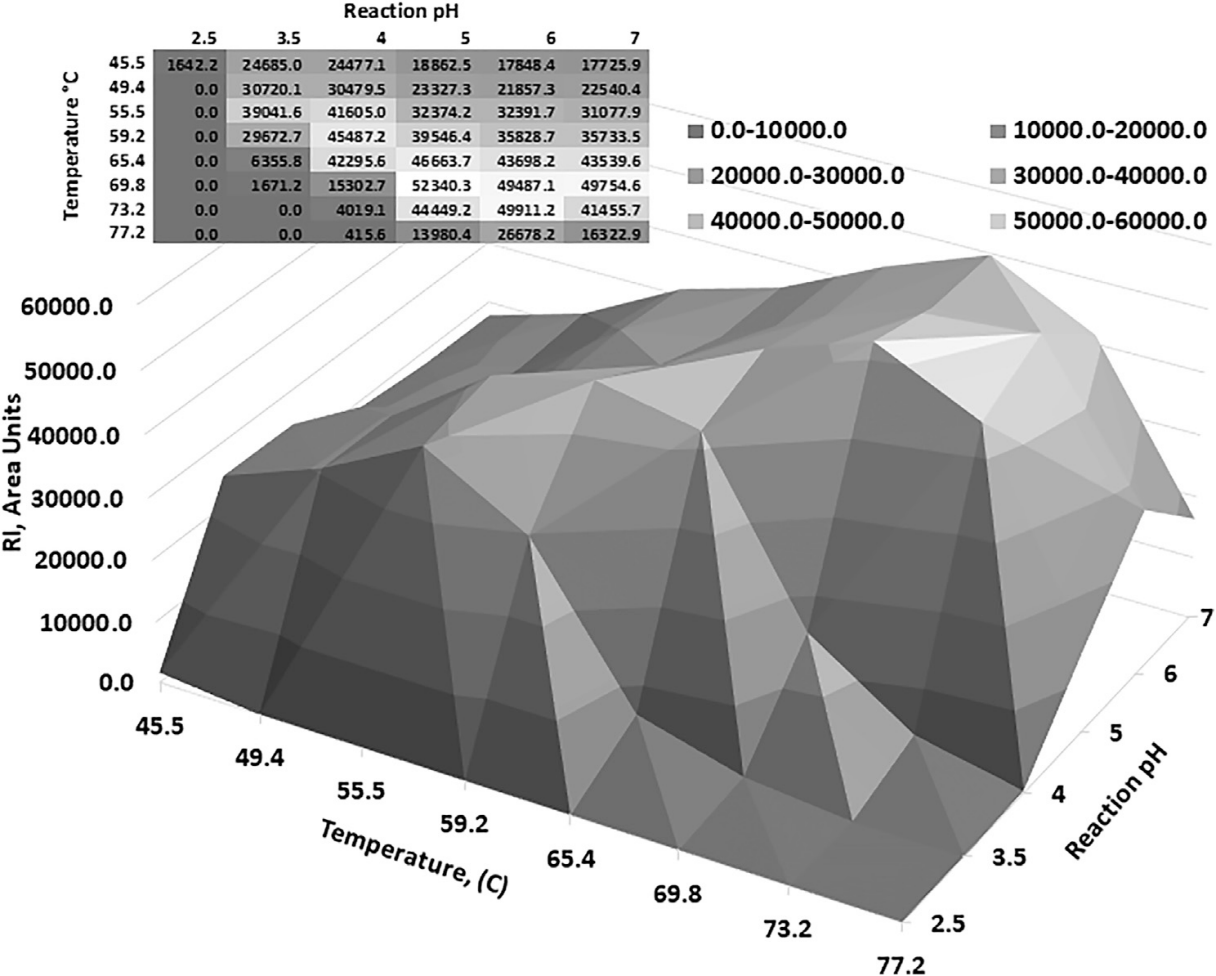
To better understand the relationship between pH and temperature optimum, a factorial study was performed. The results indicate that the *Ac*Xbh30A-CD SEP is thermostable up to about 70 C and the optimum pH increased as the reaction temperature increased.

Optimum reaction temperature analysis showed that the *Ac*Xbh30A-CD SEP, over a reaction period of 10 min, functioned at elevated temperatures (Supplementary Figure S3A). Thermostability determination of *Ac*Xbh30A-CD SEP preincubation showed that neutral pH conditions were better at preserving activity than in the lower pH conditions that were found to be optimal for the 40 C reaction conditions (Supplementary Figures S3B,C). To capture a reaction-based thermostability result, a factorial study was designed to include a range of FAMM pH conditions over an extended reaction time of 2 h. The results of this study (Figure 3) clearly demonstrated that the *Ac*Xbh30A-CD SEP is thermostable over extended reaction times under more neutral reaction pH conditions. The general relationship shows that optimum activities occur at lower temperatures in the lower pH conditions and at higher temperatures in the higher pH conditions (toward neutral pH). Peak activity at pH 3.5 and 56 C was about 20% lower than the peak activity at pH 6 and 73 C.

### Additional Purification to Yield *Ac*Xbh30A-CD GEP

For protein crystallization and kinetic studies, *Ac*Xbh30A-CD was subjected to additional purification (Supplementary Figure S4). Rather than an IMAC single-step elution, a twenty-column-volume gradient was performed. This resulted in two peaks which significantly overlapped due to the high level of column saturation. This indicated that while SDS-PAGE (Supplementary Figure S2) showed a single band of the correct molecular weight, the IMAC SEP consists of an undefined *Ac*Xbh30A-CD multimeric agglomeration. Gel filtration sizing (Superose 6 10/300 GL) and purification (Superdex, HiLoad 16/600, 200 PG) as well as anion exchange (Mono Q 4.6/100 PE) separations further support this finding (Supplementary Figure S4). The leftmost IMAC peak (Peak A) ran to size (no quaternary structure) as a well-defined peak on a calibrated Superose 6 column and also on the Superdex column used for purification. The rightmost IMAC peak (Peak B) on both gel filtration columns ran as a much larger (earlier eluting) poorly defined peak which overlapped the void volume. Similarly interesting results were found when using the Mono Q column. Peak A was a very sharp peak which eluted earlier than Peak B, which was again ill-defined, tighter-binding (eluted later), and well-resolved from Peak A. All peaks yielded the same approximately 50 kDa band in SDS-PAGE analysis (Supplementary Figure S4F). For crystal screening, the GEP was obtained from additional Peak A purification using gradient IMAC, Mono Q, and Superdex chromatography. The *Ac*Xbh30A-CD GEP was concentrated to approximately 37 mg/ml and submitted for crystallization screening at Argonne National Laboratory, Advanced Protein Characterization Facility. An initial crystal was obtained which diffracted to less than 2Å but was not successfully phased through molecular replacement methods.

Thermostability characteristics of the *Ac*Xbh30A-CD GEP were confirmed using a pNP-X_2_ reaction following a preincubation of the enzyme over time at anticipated inactivation temperatures resulting from the *Ac*Xbh30A-CD SEP studies (Supplementary Figure S3D). From these results, the *Ac*Xbh30A-CD GEP appears to be relatively stable at 68 C for 24 h. Following this, unless otherwise indicated, biochemical studies were performed at 65 C in 30 mM pH 4.0 sodium acetate buffer containing BSA at 0.02 mg/ml. For the *Ac*Xbh30A-CD GEP, the reaction pH optimum is similar to that determined for the AcXbh30A-CD SEP (data not shown).

### Further Analysis of Xylobiohydrolase Specificity

In light of a recently characterized, distantly related GH30-7 fungal XBH from *Acremonium alcalophilum* (*Aa*Xyn30A) which was shown to have detectable endoxylanase activity (Šuchová et al., 2020b) at high enzyme loadings (>5 µM), we sought to establish an empirical understanding of enzyme concentration vs. function for *Ac*Xbh30A-CD. It was established that approximately 5 μg/ml (100 nM) of *AcXbh30A*-CD could completely convert 5 mM X_4_ to X_2_ in 15 min at 60 C (data not shown). Following this initial observation, we sought to determine enzyme stringency by examining *Ac*Xbh30A-CD at 2 μg/ml (40 nM) and 20 μg/ml (400 nM) along with reaction times of 15 min or 5 h to obtain the effective relative enzyme capacity of 1x (2 μg/ml for 15 min), 10x, 20x, and 200x (20 ug/ml for 5 h).

**FIGURE 4:**
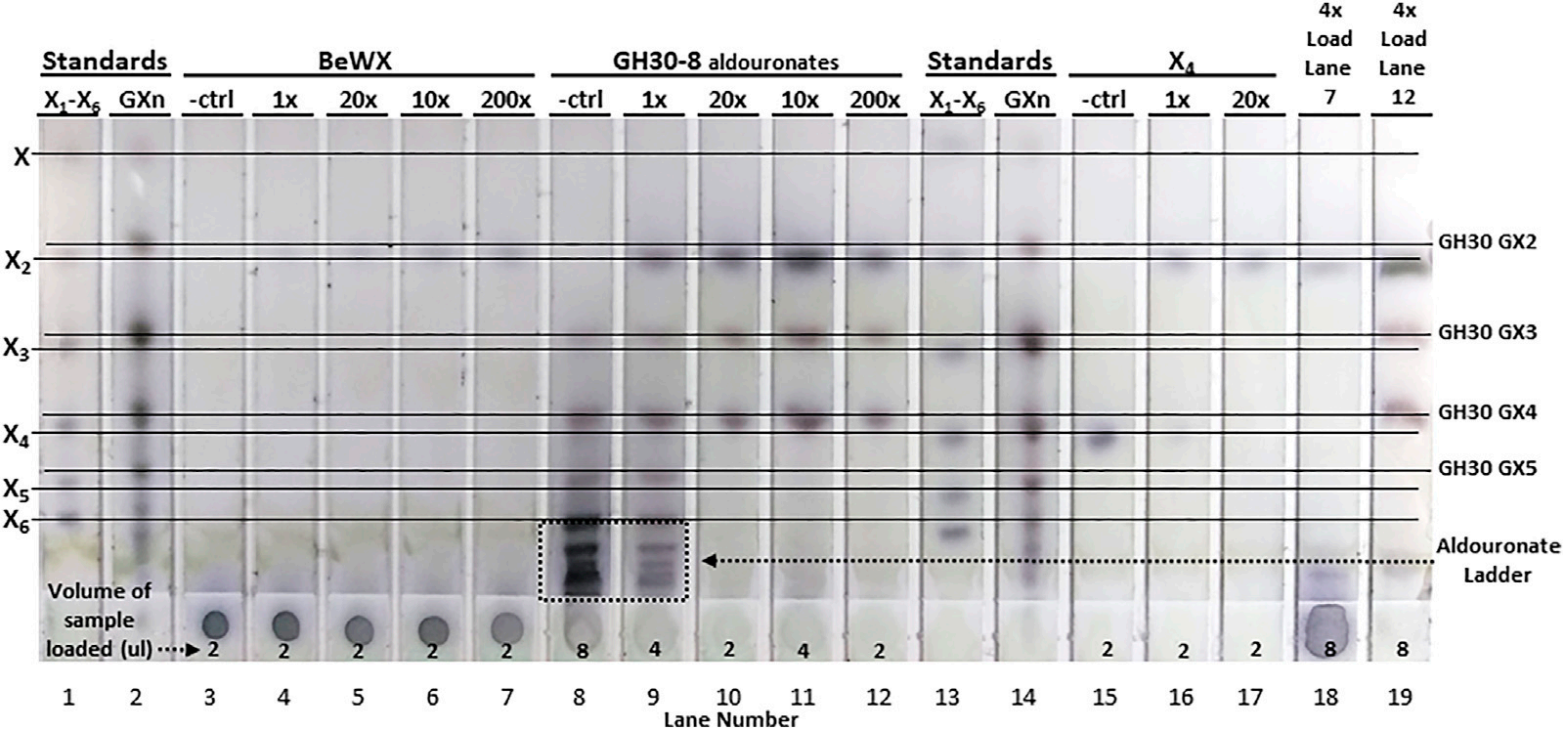
TLC analysis showing hydrolysis products of *Ac*Xbh30A-CD GEP digestion of BeWX, an enriched GH30-8 aldouronate preparation and X_4_. Using a reaction concentration of *Ac*Xbh30A-CD GEP which empirically was determined to visually represent significant X_4_ hydrolysis to X_2_ (2 ug/ml for 15 min, 1x), enzyme concentration was increased 10-fold and reaction time increased to obtain 10x, 20x, and 200x increased relative enzyme capacity. In treatment of BeWX, only a light spot for X_2_ was observed which reached approximately full intensity with the 10x enzyme condition. Complete conversion of the GH30-8 aldouronate was observed with the 10x enzyme level, and up to 200x treatment did not result in secondary activities detectable by TLC. As expected, *Ac*Xbh30A-CD GEP at 1x almost completely converted 5 mM X_4_ to X_2_. Increased load volumes (4x) of the 200x enzyme capacity reactions of BeWX and the GH30 aldouronate preparation did not reveal other hydrolysis products.

For hydrolysis of BeWX, X_2_ was the only sugar observed having low intensity on the TLC for the 10x, 20x, and 200x enzyme levels. This indicates that *Ac*Xbh30A-CD can only generate a limited amount of X_2_ from polymeric glucuronoxylan (Figure 4). This is most likely because iterative hydrolysis of a terminus will eventually be interrupted by the occurrence of a GA substitution and a lack of appreciable endoxylanase activity to generate additional non-reducing termini. The sample origin spot was visually unchanged relative to the no enzyme control for this reaction. To elucidate the directionality of *Ac*Xbh30A, a GH30-8–generated aldouronate mixture was used as the substrate (Supplementary Figures S1A–C) (St John et al., 2006a). This substrate is ideal for determination of the directionality of XBH function and, given its larger oligomeric size and complexity, may also identify any secondary activities displayed by *Ac*Xbh30A. As can be observed in Figure 4, with this aldouronate preparation at 10 mg/ml and *Ac*Xbh30A-CD at 1x, the aldouronate ladder is partially processed. The use of *Ac*Xbh30A-CD at 10x, 20x, and 200x with this substrate showed complete conversion of the aldouronate ladder to X_2_ and two aldouronates. By TLC comparison with aldouronate standards, these two limit-product aldouronates have the same mobility as aldotriuronate and aldotetrauronate. Given the strict release of X_2_ by *Ac*Xbh30A, these two aldouronates can only result from the non-reducing terminal release of X_2_ from even- or odd-numbered GH30-8 aldouronates, respectively. They must therefore be of the aldouronate substitution arrangement having the nomenclature GX and XGX (Supplementary Figures S1E,F, respectively) (Fauré et al., 2009).

This result indicates that the substitution of an α-1,2-linked GA moiety does not prevent binding and hydrolysis at the glycosidic bond most proximal to the GA-substituted xylose. However, the requirement for successful hydrolysis is the recognition of a non-reducing terminal xylobiose extension, which then yields GX. Otherwise, a single xylose remains yielding XGX. These results and further deductions agree with known GH30 protein structure and xylan binding interactions concerning the orientation of xylose positioned in the +1 subsite (Freire et al., 2016). No other products were detected from these reactions. Hydrolysis of X_4_ with 10x more enzyme resulted in only X_2_. To fully assess the potential for secondary hydrolysis products other than X_2_ (possible endoxylanase activity), the TLC spot load volume for the 200x reactions of BeWX and GH30-8 aldouronates were increased 4x. For these, only a very light, large xylooligosaccharide smear is detected along with X_2_, GX, and XGX.

### Specific Activity and Kinetic Analysis of *Ac*Xbh30A-CD GEP

Specific activity and kinetic analysis of *Ac*Xbh30A-CD GEP was performed at the optimized functional parameters of 65 C and pH 4.0. The result of xylooligosaccharide hydrolysis to X_2_ by HPLC is from duplicate reactions with each being injected three times. The overall average was used for rate calculations. The results (Table 1) indicate that only relatively minor rate variations are evident between the hydrolysis of X_3_–X_6_. The highest specific activity of 182.1 U/mg was observed for X_3_. X_6_ has the next highest at 165.1 U/mg. X_4_ and X_5_ are slightly lower at 157.7 and 150.2 U/mg, respectively. 

**TABLE 1:** Specific activity measurements of *Ac*Xbh30A-CD and homologous enzymes.

Enzyme	Substrate	Specific activity, U/mg (% error)
***Ac*Xbh30A-CD** ^a^	X_3_	182.09 ± 2.78 (1.52)
	X_4_	157.68 ± 1.31 (0.83)
	X_5_	150.21 ± 2.56 (1.70)
	X_6_	165.06 ± 4.60 (2.79)
	pNP-X_2_	170.87 ± 3.71 (2.17)
***Ac*Xbh30A-CD** ^b^	pNP-X_2_	31.90 ± 0.72 (2.26)
***Pc*Xbh30A** ^b^ ^,^ ^c^	pNP-X_2_	10.12 ± 0.25 (2.44)
***Pp*Xbh30A** ^b^ ^,^ ^d^	pNP-X_2_	3.20 ± 0.05 (1.57)

a *Ac*Xbh30A-CD reactions were performed using 30 mM sodium acetate of pH 4, 0.02 mg/ml BSA, xylooligosaccharides at 10 mM, or pNP-X_2_ at 2 mM (ca. 17 x *K*_*M*_) at 65 C for 10 min.

b For comparison, reactions were performed as superscript “a” above, but at 37 C.

c *Pseudobacteroides cellulosolvens* GH30 annotated enzyme (*Pc*Xbh30A) with UniProt accession number A0A0L6JSW0, as *Pc*Xbh30A shares 74.8% amino acid identity with *Ac*Xbh30A.

d *Paenibacillus psychroresistens* GH30 annotated enzyme (*Pp*Xbh30A) with NCBI GenBank accession number WP_162463230. Limited TLC-based reaction optimization indicated that this GH30 xylobiohydrolase from a psychrophilic bacterium functions at 37 C, and thus, this defined the reaction temperature used for the specific activity determination for the three novel xylobiohydrolases.

Kinetic evaluation using pNP-X_2_ concentration–dependent response curves was performed with three separate experiments (Table 2). Importantly, the specific activity calculated from these same data (2 mM pNP-X_2_ data point) showed that this substrate provided a reasonable functional parallel to the natural XOS given that its observed rate of 170.9 U/mg is similar (Table 1). This may indicate that the binding energy of the aromatic pNP group into the +1 (and possible +2) subsite is similar to that of a xylosyl group. From the kinetic studies, *Ac*Xbh30A-CD was determined to have a *K*_*M*_ of 116 μM and a *V*_max_ of 183 U/mg resulting in a *k*_*cat*_ of 153/s with an enzyme efficiency constant of 1,312/s mM (Table 2). X_2_ was also considered for product inhibition using a standard competitive inhibition model. The finding indicates a *K*_*i*_ of 2.4 mM. This is similar to the reported cellobiose inhibition (*K*_*i*_, 1.6 mM) of the processive cellobiohydrolase Cel7A in hydrolysis of bacterial cellulose (Gruno et al., 2004). Lastly, we assessed the tendency of *Ac*Xbh30A-CD to act processively in the hydrolysis of X_6_ using HPLC. The measurement of X_4_ at appreciable levels during the reaction indicated that its function is not processive (Supplementary Figure S5).

**TABLE 2:** Kinetic constants of *Ac*Xbh30A in the hydrolysis of pNP-X_2_.

Kinetic constant	*Ac*Xbh30A pNP-X_2_ kinetic values^a^ ^,^ ^b^
*K*_*M*_	116.4 ± 3.23 μM
***V***_***max***_	183.1 ± 1.13 U mg^−1^
***k***_***cat***_	152.7 s^−1^
***K***_***cat***_ ***/K***_***M***_	1,311.6 s^−1^ mM^−1^
***K***_***i***_ **(X**_**2**_ **)** ^c^	2.4 mM

a Standard assay consisted of 100 μl reactions containing 30 mM sodium acetate pH 4.0, 0.02 mg/ml BSA, variable concentrations of pNP-X_2_ in the range of 30 μM–4 mM, and 5 ng of *Ac*Xbh30A-CD GEP enzyme which was incubated at 65 C for 10 min. The reaction was stopped and developed by addition of 300 μl of 200 mM sodium carbonate and measured at 405 nm. Baseline corrected data were converted to concentration of pNP using its reported mM extinction coefficient (ε = 18.4).

b For substrate concentration–dependent kinetics, three separate assays were performed. A least-squares fit of the non-linear Michaelis–Menten enzyme kinetics model in GraphPad Prism 8 was used to obtain kinetic values of the three curves as a group. The error is reported as the standard error of parameters, and the standard error of fit was 2.532 U/mg.

c Product inhibition was studied by inclusion of X_2_ in the standard pNP-X_2_ assay at concentrations of 0.5, 1, 2, and 4 mM. No X_2_, 0.5 mM X_2_, and 1.0 mM X_2_ datasets were performed at least twice, while the datasets for 2 and 4 mM X_2_ were only performed a single time. A robust fit of the non-linear competitive enzyme inhibition model in GraphPad Prism 8 was used to obtain the inhibition constant (*K*_*i*_).

### Analysis of Xylobiohydrolase Specificity

The recent publications by Šuchová et al. address a detected endoxylanase activity for both primary xylobiohydrolases *Aa*Xyn30A (Šuchová et al., 2020b) and *Ac*Xbh30A (Šuchová et al., 2020a). In light of our earlier efforts to confirm functional specificity for *Ac*Xbh30A, we felt compelled to reproduce these results: *Ac*Xbh30A (*Hc*Xyn30A as it was previously designated), we confirm, does have detectable endoxylanase activity under the same conditions employed by Šachová et al. However, to obtain this outcome, GXn hydrolysis reactions were conducted for extended periods with 2.4 uM of enzyme (5.4 uM in the case of *Aa*Xyn30A). With respect to the data reported in this work, and keeping with the same enzyme capacity factor concept, the endoxylanase activity detected by Šuchová was obtained with the equivalent of ca. 6,000x, the base condition of 2 ug/ml *Ac*Xbh30A-CD for 15 min (1x). Given this analysis, we posit that the reported endoxylanase activity is not reflective of biologically relevant specificity but may be of value in the isolation of novel substituted xylooligosaccharides.

### Phylogenetic Placement of *Ac*Xbh30A

**FIGURE 5:**
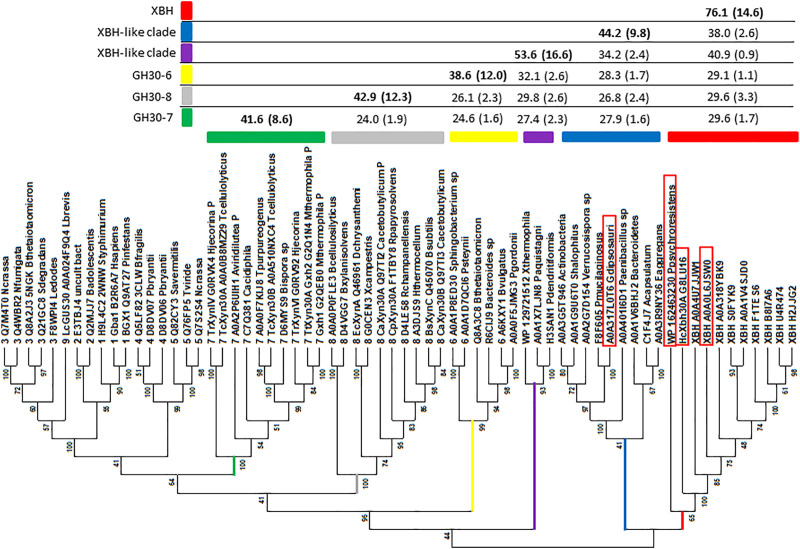
Phylogenetics analysis distinguishing the GH30 subfamily branches and highlighting the new XBH subfamily (red branch). The sequence set includes all subfamily member sequences determined through published reports or protein structure database deposits collected from CAZy as well as a group homologous to *Ac*Xbh30A. Sequences were trimmed to consist of only the (β/α)_8_ + β complete GH30 domain (St John et al., 2010). Sequences were aligned using MAFFT (Katoh and Toh, 2008) with the G-INS-1 strategy and imported into MEGA X (Kumar et al., 2018) where evolutionary history was inferred by using the maximum likelihood method and the LG substitution model (Le and Gascuel, 2008). The bootstrap consensus tree inferred from 100 replicates (Felsenstein, 1985) is taken to represent the evolutionary history of the taxa analyzed (Felsenstein, 1985). The percentage of replicate trees in which the associated taxa clustered together in the bootstrap test (100 replicates) is shown next to the branches (Felsenstein, 1985). Initial trees for the heuristic search were obtained automatically by applying neighbor-join and BioNJ algorithms to a matrix of pairwise distances estimated using a JTT model and then selecting the topology with a superior log likelihood value. A discrete gamma distribution was used to model evolutionary rate differences among sites [three categories (+G, parameter = 2.5225)]. The rate variation model allowed some sites to be evolutionarily invariable ([+I], 0.28% sites). This analysis involved 66 amino acid sequences. There were a total of 878 positions in the final dataset. These sequences also included GH30-7 (green branch), GH30-8 (gray branch), and GH30-6 (yellow branch), and two XBH-like branches (purple and blue) were analyzed using PRSS (Pearson, 1998). The results of percent identity are tabulated to distinguish levels of rough similarity cut-offs between GH30-6, -7, and -8 subfamilies, the unknown XBH-like clades, and the new XBH subfamily (red). Enzymes boxed in red were evaluated for biochemical function following DNA cloning, protein expression, and IMAC purification.

Original GH30 subfamily classification proposed the existence of eight subfamilies, several of which had existing functional annotation assignments. At the time, large-scale amino acid phylogenetic analysis showed that subfamily members shared greater than ca. 30% identity, with comparison between subfamilies dropping to less than ca. 28% identity (St John et al., 2010). Since this time, a new functional subfamily, GH30-9, has been delineated from its nearest neighbor GH30-3 (Sakurama et al., 2013). *Ac*Xbh30A is a member of a clade with members sharing at least 46% identity. To verify that this represented a collection of prokaryotic XBH enzymes, we cloned and expressed an enzyme from *Pseudobacteroides cellulosolvens* (UniProt accession No. A0A0L6JSW0, *Pc*Xbh30A) that shares 74.8% identity with *Ac*Xbh30A and an enzyme from *Paenibacillus psychroresistens* (GenBank accession No. WP_162463230, *Pp*Xbh30A) which establishes a boundary position at the edge of the clade (Figure 5) and shares ca. 50% identity with *Ac*Xbh30A. Both enzymes have XBH activity within 10-fold (lower) of *Ac*Xbh30A (Table 1). These enzymes were not rigorously optimized for function regarding temperature, pH, etc., and comparison reactions with *Ac*Xbh30A were all performed at 37 C to provide more comparable results. Importantly, the XBH clade, except for *Pp*Xbh30A, represents enzymes solely from the bacterial order Clostridiales. The phylogenetic nearest neighbor consists of protein sequences from a more diverse collection of bacteria and shares greater than ca. 34% identity with *AcXbh30A*. In this neighboring group, the highest sequence identity to *AcXbh30A* is 43.9%. Explorations into the enzymatic activity of this adjacent clade are ongoing, but initial indications are that GH30 from *Gracilibacillus dipsosauri* (UniProt accession No. A0A317L0T6) displays endoxylanase activity which will be reported elsewhere (*Gd*Xyn30A). No XBH activity is detected with this enzyme.

As discussed above, at the time of the original GH30 subfamily assignment, it was determined that members of each of the detected subfamilies shared greater than 30% identity with identity levels between subfamilies dropping to below 28% (St John et al., 2010). For instance, PRSS analysis of all GH30-7 enzymes against all GH30-8 enzymes used in Figure 5 results in an average identity of 24.5% (SD 1.6%). A similar comparison between the newly delineated GH30-9 subfamily (Sakurama et al., 2013) and the larger more diverse GH30-3 subfamily gives an average identity of just 30.1% (SD 0.9%). The ultimate goal of the CAZy database is the functional annotation of enzyme groups, and the exo-acting β-glucuronidase function of GH30-9 is functionally and likely structurally (at least in the active site) different from the endo-β-1,6-glucanase function annotated into GH30-3. An amino acid sequence identity screen, using all the members of each clade in the dataset (Figure 5), shows that while the level of homology between the XBH clade and its neighboring clade hovers around ca. 40%, the identity levels with all other members of GH30-6, -7, and -8 are in the range of 30%. Based on this observation, it seems appropriate that given its unique biochemical function, *Ac*Xbh30A defines a new GH30 subfamily. Communication with curators of the CAZy database has indicated acceptance of this proposed subfamily as GH30, subfamily 10 (personal communication during manuscript revision).

### Co-Digestion of GXn Using Canonical GH30-8 Enzymes and the New GH30 Xylobiohydrolases

**FIGURE 6:**
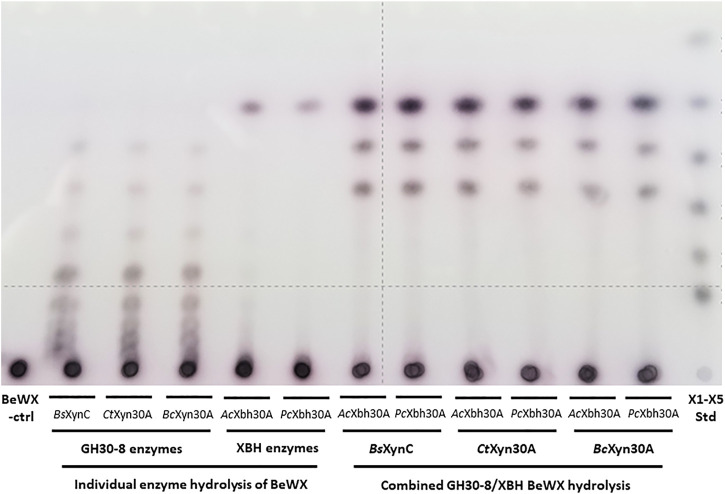
TLC analysis verifying function of three canonical functioning GH30-8 glucuronoxylanases *Bs*XynC (St John et al., 2006a), *Ct*Xyn30A (St John et al., 2017), and *Bc*Xyn30A (unpublished) and the newly identified xylobiohydrolase *Ac*Xbh30A-CD [this work and Šuchová et al. (2020a)] as well as its homolog *Pc*Xbh30A (this work) in the hydrolysis of polymeric glucuronoxylan. When combined, these enzymes convert glucuronoxylan to the three products X_2_, GX, and XGX (Supplementary Figures S1D–F, respectively).

In consideration of the complete conversion of the GH30-8 aldouronate preparation to X_2_, GX, and XGX, it was considered of value to broadly examine the utility of XBHs to enable the complete conversion of GXn to the three defined products X_2_, GX, and XGX in a two-enzyme system. To verify the relationship between these two enzymes, we considered the XBHs *Ac*Xbh30A-CD and *Pc*Xbh30A (from *P. cellulosolvens*, see above) as well as three different canonical GH30-8 endoxylanases. These GH30-8 members include *Bs*XynC from *Bacillus subtilis* (St John et al., 2006a), *Ct*Xyn30A from *Clostridium thermocellum* (St John et al., 2017), and *Bc*Xyn30C from *Bacteroides cellulosilyticus* (unpublished). All three GH30-8 enzymes functioned as anticipated yielding a ladder of aldouronates, and when combined as co-digestions with either of the two tested XBHs described here, the predicted three defined limit products are observed (Figure 6). This shows that the hydrolytic product observations made here likely extrapolate to all canonical GH30-8 enzymes when coupled to this new GH30 XBH subfamily.

## Conclusion

The *Ac*Xbh30A enzyme was originally observed as being a prominent component of the *A. clariflavus* cellulosome during growth on cellulosic substrates (Artzi et al., 2015). The functional understanding of this enzyme as a xylobiohydrolase coupled with its significant presence in the cellulosome highlights a novel strategy of lignocellulose deconstruction and utilization by this cellulosome-producing bacterium and further lends potential in the development of synthetic cellulosome assemblies for lignocellulose bioconversion (Kahn et al., 2019; Ding and Bayer, 2020). From these studies, *Ac*Xbh30A is shown to function as an NRT-specific XBH which when utilized optimally generates no detectable secondary products. *Ac*Xbh30A has a wider functional pH range then commonly observed for glycoside hydrolases and as an apparent enzyme monomer is stable over extended time periods up to 65 C. Coupling enzymes of this new XBH GH30 subfamily with the specificity of the GH30-8 glucuronoxylanases in the processing of GXn yields the prebiotic sugar X_2_ and two valuable aldouronates of defined structure. The same XBH–GH30-8 enzyme system, if presented with more complex xylan types that also contain GA substitutions, has the potential to yield a multitude of novel substituted oligoxylosides.

## Data Availability

The original contributions presented in the study are included in the article/Supplementary Material, and further inquiries can be directed to the corresponding author.

## Footnotes

1
https://www.uniprot.org/

2
https://blast.ncbi.nlm.nih.gov/Blast.cgi

3
http://www.cazy.org/GH30.html

4
https://mafft.cbrc.jp/alignment/server/

5
https://fasta.bioch.virginia.edu/fasta_www2/fasta_list2.shtml

6
https://www.nzytech.com/products-services/cazymes/glycoside-hydrolases/glucuronoxylanases/cz0916/

## Abbreviations

Araf, α-L-arabinofuranosyl; BSA, bovine serum albumin; BeWX, beechwood xylan; BiWX, birchwood xylan; FAMM, acronym for the buffer used in this research that is composed of 30 mM each of sodium formate, sodium acetate, MES (2-(N-morpholino)ethanesulfonic acid), and MOPS (3-(N-morpholino)propanesulfonic acid); GA, glucuronic acid; GXn, glucuronoxylan; GEP, gradient elution product; X2, xylobiose; X3, xylotriose; X4, xylotetraose; X5, xylopentaose; X6, xylohexaose; pNP, p-nitrophenol; pNP-X2, p-nitrophenol xylobioside; SEP, step elution product; XOS, xylooligosaccharide.
