# Supplementary material for: A New Subfamily of Glycoside Hydrolase Family 30 with Strict Xylobiohydrolase Function
Source: Front Mol Biosci. 2021 Sep 7;8:714238. doi: 10.3389/fmolb.2021.714238 (PMC8453022; doi:10.3389/fmolb.2021.714238)
Supplement: Supplementary file 1 [file DataSheet1.pdf]

Supplemental Figure 1

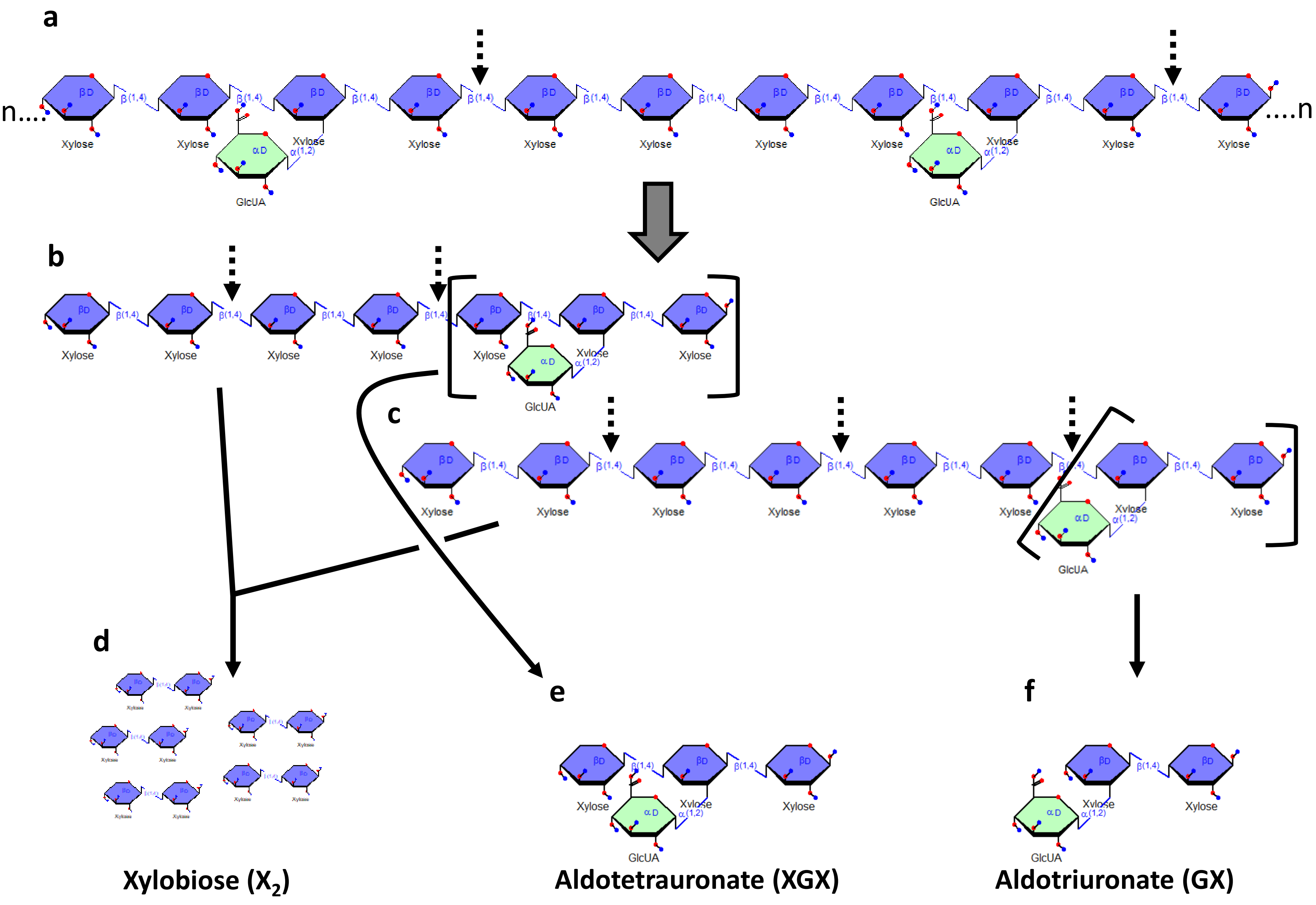

Supplemental Figure 1:

Pictorial representation of glucuronoxylan (GXn) and its processing by the enzymes considered in this work. Alpha-1,2-linked glucuronic acid (GA) moieties of GXn serve as canonical functioning GH30-8 endoxylanase recognition sites (a, dashed arrows) (St John et al., 2006). Limit hydrolysis of GXn by these enzymes results in a pool of variably sized aldouronates characteristic of the nature of the substitution of GA along the xylan chain, which, in the case of hardwood GXn is thought to be random (Jacobs et al., 2001). GH30-8 aldouronates are known to consist of variably sized xylooligosaccharides substituted near the reducing terminus with a single GA on the penultimate xylose (Vršanská et al., 2007). Given this aldouronate product pool variability, both even and odd numbered aldouronates are represented (b, c). Hydrolysis of this population of specifically structured aldouronates using the new *Ac*Xbh30A xylobiohydrolase reveals the specificity of non-reducing terminus directionality and also how this enzyme accesses GA-substituted regions of xylan. Given the TLC analysis of *Ac*Xbh30A hydrolysis of these GH30-8 aldouronates (Fig. 4), only xylobiose (X_2, d), aldotetrauronate (XGX, e) and aldotriuronate (GX, f) are produced. This indicates that *Ac*Xbh30A has access to the glycosidic bond most proximal to the GA-substituted xylose and therefore based on the known protein structure relationships for this enzyme family (Freire et al., 2016; St John et al., 2011; Urbániková et al., 2011) that the GA-substituted xylose is readily accommodated in the +1 aglycone subsite.

Supplemental Table 1: General information and cloning aspects of the enzymes considered in this work

Organism	Enzyme Designation	Database: Accession	Native modular Structure [a]	Expression product: N-term … C-term [b]	Expressed protein characteristics [a, b]
Acetivibrio clariflavus	*Ac*Xbh30A	UniProt: G8LU16	SPI/GH30/DCKI	MG(H6)ASTV … PVEQ [c]	H6/GH30/DCKI
Acetivibrio clariflavus	*Ac*Xbh30A	UniProt: G8LU16	SPI/GH30/DCKI	MASTV … VEQLE(H6) [d]	GH30/DCKI/H6
Acetivibrio clariflavus	*Ac*Xbh30A-*CD*	UniProt: G8LU16	SPI/GH30/DCKI	MASTV … SASLE(H6) [d]	GH30/H6
Pseudobacteroides cellulosolvens	*Pc*Xbh30A-*CD*	UniProt: A0A0L6JSW0	SPI/GH30/DCKI	MASTV … VGALE(H6) [d]	GH30/H6
Paenibacillus psychroresistens	*Pp*Xbh30A	GenBank: WP_162463230	SPI/GH30	MADGI … RNDLE(H6) [d]	GH30/H6
Gracilibacillus dipsosauri	XBH similar	UniProt: A0A317L0T6	SPI/GH30/CBM6/CBM4_9	MAKTA … EFELE(H6) [d]	GH30/H6

[a] SPI, a secretion leader sequence with a signal peptidase type 1 cleavage site; GH30, a glycoside hydrolase family 30 domain; DCKI, type 1 dockerin domain; CBM6, family 6 carbohydrate binding module; CBM4_9, family 4_9 carbohydrate binding module

[b] Enzymes cloned to include N-terminal and C-terminal bounded amino acid sequences. H6, 6x-His Tag

[c] (Artzi et al., 2015)

[d] This work

Supplemental Figure 2

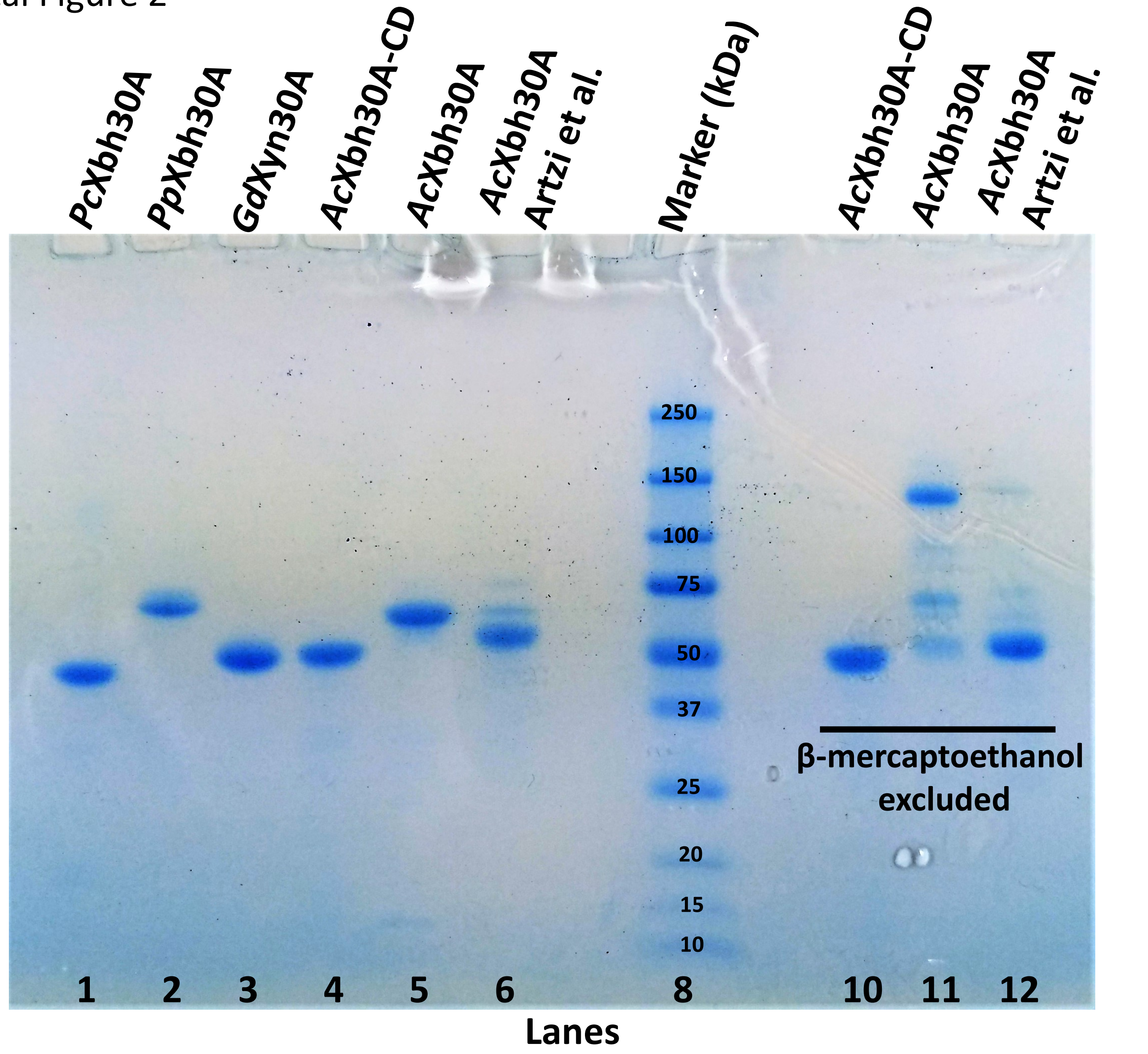

Supplemental Figure 2:

SDS-PAGE size and purity analysis of the IMAC purified *Ac*Xbh30A expression products along with its protein homologs considered in this work. Protein samples on the left side of the gel were processed as is normal for denaturing gel electrophoresis, while for the protein samples on the right the reducing agent β-mercaptoethanol was exclude. Lanes 1-3 indicates the relative purity following IMAC purification and apparent molecular weight of the *Ac*Xbh30A homologs that were considered in this work. The IMAC purified expression product *Ac*Xbh30A-CD (Lane 4) and *Ac*Xbh30A (Lane 5) showed apparent MW as anticipated from their theoretical masses. One reason for the recloning of the original N-terminal His-Tag version of *Ac*Xbh30A (Lane 6) (Artzi et al., 2015) can be seen from the two bands that occur with the expression product. The upper minor band runs to the size of the full two domain (GH30/dockerin) product as compared with Lane 5 and the major band does not run as small as *Ac*Xbh30A-CD in Lane 4. The existence of two bands is possibly a result of proteolysis of the two-domain protein. In the recently published report concerning the same enzyme addressed here (Šuchová et al., 2020), they characterized a commercially available version of this enzyme which from the commercial product information appears similar when analyzed by SDS-PAGE (NYZTech product webpage). Consideration of the full length *Ac*Xbh30A expression product without the reducing agent β-mercaptoethanol showed that it runs as a dimer indicating a disulfide linkage occurs (Lane 11) which must involve Cys516 located in the C-terminal dockerin domain. To a much lesser extent this dimeric expression product can also be detected in the original N-terminal His-Tag version of *Ac*Xbh30A.

Supplemental Figure 3

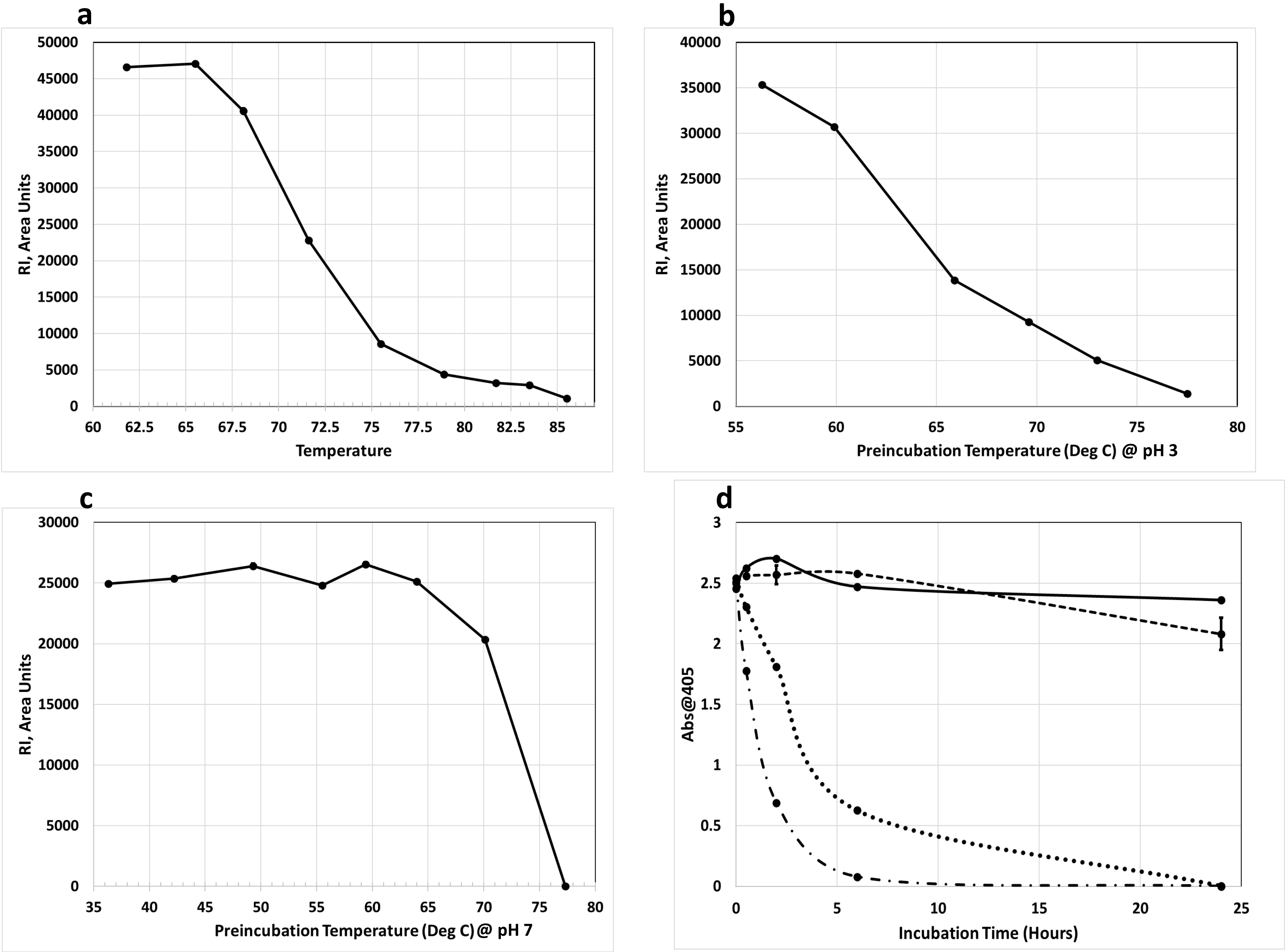

Supplemental Figure 3:

Optimum reaction temperature (a) and thermostability analysis (b-c) were performed using *Ac*Xbh30A-CD SEP or the more highly purified *Ac*Xbh30A-CD GEP preparation (d). Activity was measured by following the hydrolysis of X_4 to X_2 at 40 °C in pH 3.75 FAMM buffer by HPLC. Optimum reaction temperature was determined for a 10-minute reaction (a). Thermostability was assayed following preincubation of 50 µg/ml *Ac*Xbh30A-CD SEP for 2.5 hr at pH 3 (30 mM glycine) (b) and pH 7 (20 mM Tris base) (c). Residual activity was measured as above, but for 20 min. To verify enzyme thermostability of the GEP enzyme preparation, 5 µg/ml *Ac*Xbh30A-CD GEP was preincubated in 30 mM sodium acetate, pH 6.0 with BSA at 0.02 mg/ml at selected high temperatures, sampled over an extended time frame and assayed using the standard pNP-X_2 assay (d). From the top solid line showing *Ac*Xbh30A-CD thermostability at 64.8 °C, increasing temperature incubation conditions are shown with dashed lines in descending order of 68.3 °C, 70.6 °C and 71.4 °C, respectively. For these studies, only the 68.3 °C temperature study was repeated and therefore error bars are shown in graph for this temperature.

Supplemental Figure 4

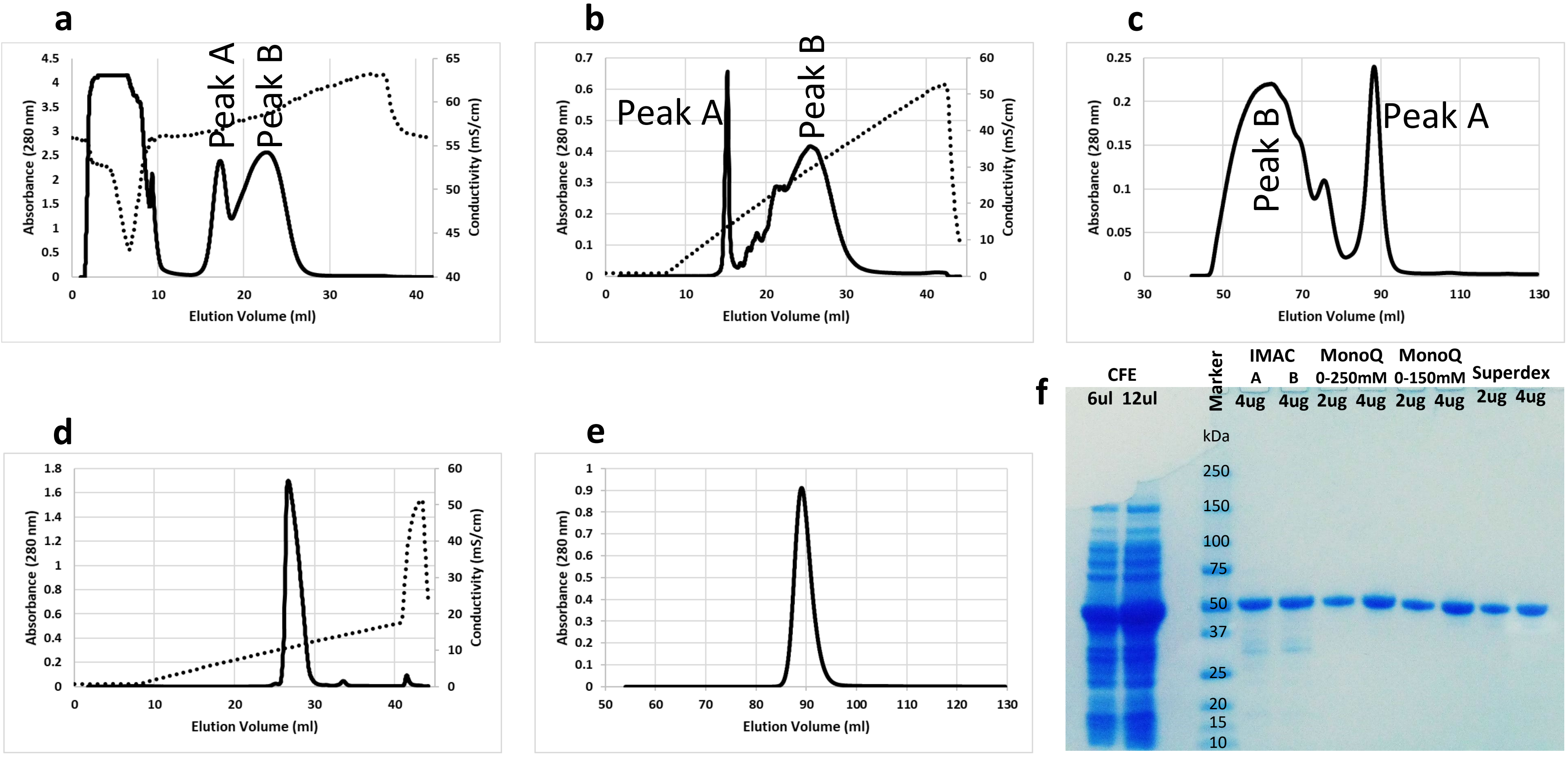

Supplemental Figure 4:

Chromatograms of additional purification steps to yield *Ac*Xbh30A-CD GEP which highlight the need for further definition of the solution state of this enzyme, its impact on functional characteristics and enzyme application. Figure (a), (b) and (c) shows the outcome of reinjection of the *Ac*Xbh30A-CD SEP on a gradient IMAC, Mono-Q and Superdex column, respectively with each showing both purification forms of *Ac*Xbh30A-CD. Peak assignment for (b) and (c) result from peak isolation following (a) and subsequent analysis of Peak A and Peak B by Mono Q or Superdex. In the isolation of Peak A for protein crystallography purposes, Mono Q separation was optimized to resolve over a 0-150 mM sodium chloride, 20 column volume gradient (d) and polishing was performed using a Superdex column (e). SDS-PAGE analysis (f) of *Ac*Xbh30A-CD GEP purification steps showing that all chromatographic peaks represent the apparent mass of *Ac*Xbh30A-CD (ca. 50 kDa).

Supplemental Figure 5

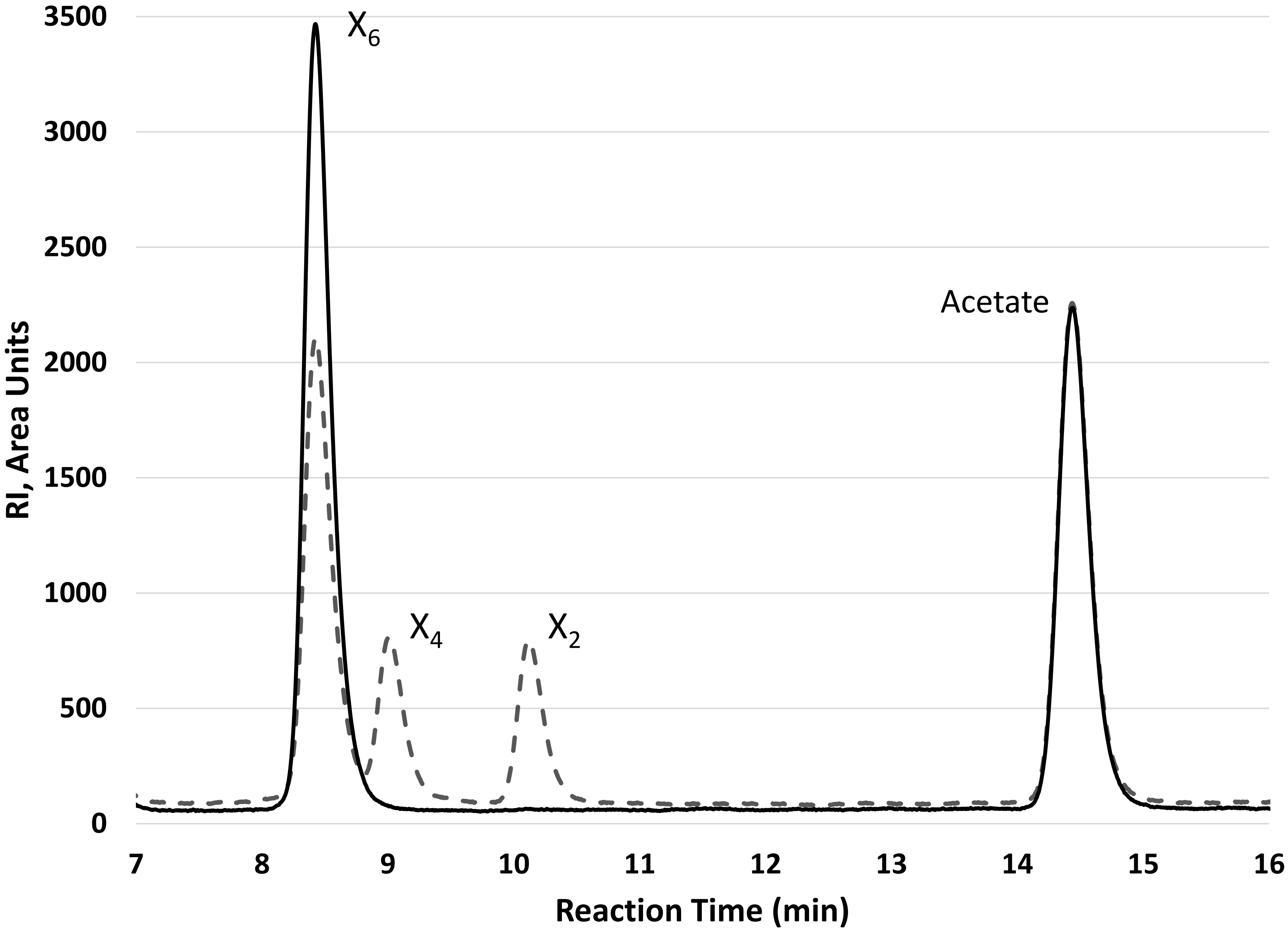

Supplemental Figure 5:

Two overlayed HPLC chromatograms showing the no enzyme control (solid, black line) and the 5-minute reaction time point (greyed, dashed line) for *Ac*Xbh30A-CD SEP hydrolysis of X_6. The reaction was intended to qualitatively determine if *Ac*Xbh30A displayed processive hydrolytic character. It was reasoned that if *Ac*Xbh30A was perfectly processive and the molar concentration of enzyme was much less then initial concentrations of X_6 then no X_4, as a reaction intermediate, would be detectable and only X_2 would be measurable. In the obtained data, using *Ac*Xbh30A-CD SEP at 100 nM, sodium acetate pH 5 at 30 mM , BSA at 0.1 mg/ml and X_6 at 1 mM, X_4 was measured being comparable to the amount of X_2 detected. These results indicate that AcXbh30A-CD displays no significant processive hydrolytic character.

Supplemental References:

Artzi, L., Morag, E., Barak, Y., Lamed, R., Bayer, E.A. 2015. Clostridium clariflavum: key cellulosome players are revealed by proteomic analysis. MBio, 6(3), e00411-15.

Freire, F., Verma, A., Bule, P., Alves, V.D., Fontes, C.M., Goyal, A., Najmudin, S. 2016. Conservation in the mechanism of glucuronoxylan hydrolysis revealed by the structure of glucuronoxylan xylanohydrolase (CtXyn30A) from Clostridium thermocellum. Acta Crystallogr D Biol Crystallogr., 72(11), 1162-1173.

Jacobs, A., Larsson, P.T., Dahlman, O. 2001. Distribution of uronic acids in xylans from various species of soft-and hardwood as determined by MALDI mass spectrometry. Biomacromolecules, 2(3), 979-990.

St John, F.J., Hurlbert, J.C., Rice, J.D., Preston, J.F., Pozharski, E. 2011. Ligand bound structures of a glycosyl hydrolase family 30 glucuronoxylan xylanohydrolase. J. Mol. Biol., 407(1), 92-109.

St John, F.J., Rice, J.D., Preston, J.F. 2006. Characterization of XynC from Bacillus subtilis subsp. subtilis strain 168 and analysis of its role in depolymerization of glucuronoxylan. J. Bacteriol., 188(24), 8617-8626.

Šuchová, K., Puchart, V., Biely, P. 2020. A novel bacterial GH30 xylobiohydrolase from Hungateiclostridium clariflavum. Applied Microbiology and Biotechnology, 1-11.

Urbániková, L., Vršanská, M., Morkeberg Krogh, K.B., Hoff, T., Biely, P. 2011. Structural basis for substrate recognition by Erwinia chrysanthemi GH30 glucuronoxylanase. FEBS J., 278, 2105.

Vršanská, M., Kolenová, K., Puchart, V., Biely, P. 2007. Mode of action of glycoside hydrolase family 5 glucuronoxylan xylanohydrolase from Erwinia chrysanthemi. Febs J., 274(7), 1666-1677.
